# Analysis of Dynamics Targeting CNT-Based Drug Delivery through Lung Cancer Cells: Design, Simulation, and Computational Approach

**DOI:** 10.3390/membranes10100283

**Published:** 2020-10-14

**Authors:** Nafiseh Sohrabi, Afshar Alihosseini, Vahid Pirouzfar, Maysam Zamani Pedram

**Affiliations:** 1 Department of Chemical Engineering, Central Tehran Branch, Islamic Azad University, Tehran, Iran; nafsoh@gmail.com (N.S.); v.pirouzfar@iauctb.ac.ir (V.P.); 2 Faculty of Electrical Engineering, K.N. Toosi University of Technology, Tehran 16315-1355, Iran

**Keywords:** carbon nanotube (CNTs), molecular dynamics simulation, cell membrane, dynamic system modeling

## Abstract

Nowadays, carbon nano (CN) structures and specifically carbon nanotubes (CNTs), because of the nanotube’s nanoscale shape, are widely used in carrier and separation applications. The conjugation of CNTs with polysaccharide, proteins, drugs, and magnetic nanoparticles provides a chance for smart targeting and trajectory manipulation, which are used in the crucial field of life science applications, including for cancer disease diagnostics and treatments. Providing an optimal procedure for delivering a drug to a specific area based on mathematical criteria is key in systemic delivery design. Trajectory guidance and applied force control are the main parameters affected by systemic delivery. Moreover, a better understanding of the tissue parameters and cell membrane molecular behaviour are other factors that can be indirectly affected by the targeted delivery. Both sides are an essential part of successful targeting. The lung is one of the challenging organs for drug delivery inside the human body. It has a large surface area with a thin epithelium layer. A few severe diseases directly involve human lung cells, and optimal and successful drug delivery to the lung for the treatment procedure is vital. In this paper, we studied functionalized CNTs’ targeted delivery via crossing through the lung cell membrane. Molecular dynamics (MD) software simulated all the interaction forces. Mathematical modelling of the cell membrane and proposed delivery system based on the relation of velocity and force has been considered. Dynamics equations for CNTs were defined in the time and frequency domain using control theory methods. The proposed delivery system consists of two main parts: crossing through the cell membrane and targeting inside the cell. For both steps, a mathematical model and a proper magnetic field profile have been proposed. The designed system provides criteria for crossing through the cell membrane within 30 s to 5 min and a translocation profile of 1 to 100 Å.

## 1. Introduction

Carbon nanotubes (CNTs) have gained full attention so far in life science applications [1,2,3,4,5,6]. These applications include separation [1,2,3,7], drug delivery [8,9,10,11,12], and hyperthermia by guiding with magnetic nanoparticles [13,14,15,16]. Because of the small size of CNTs, they can penetrate the cells non-invasively. Moreover, in the non-invasive delivery method, CNTs can be directed by exerting an external magnetic force. Furthermore, CNTs can be used as a carrier for drug delivery to cancer cells inside the body [6,17]. As CNTs can be functionalized and guided by an external force, they can also be used for targeted drug delivery to the brain [18]. Delivering drugs to the brain by exerting a non-invasive external magnetic force across the Blood Brain Barrier (BBB) provides more actuation flexibility to guide CNTs [19]. The chemotherapy magnetically controlled under Magnetic resonance imaging (MRI) is a novel technique. This technique is based on directing a magnetic nanocarrier through an MRI system where the actuation forces are driven by a magnetic field [20,21]. A critical factor in CNT delivery is the particle size. CNTs can be fabricated in different sizes ranging, from 0.8 to 20 nm. It is noteworthy that the dimensions of single-wall carbon nanotubes (SWCNTs) are in the range of 0.8 to 2 nm; therefore, in comparison with 5–20 nm multi-wall carbon nanotubes (MWCNTs), SWCNTs can enter the cytoplasm of the cell and nucleus and pass from the cell membrane easier, and they can play an essential role of a carrier to release drugs into cancer cells [22,23,24,25]. By creating a magnetic field and applying forces, magnetic CNTs can be directed to cross through the cell membrane in the application of cancer therapy [26,27]. Although clinical in vitro and in vivo modeld have been studied in cancer therapy and drug delivery [22,28,29], less attention has been paid to the advantage of CNTs for crossing through the cell membrane and providing a mathematical model for understanding the dynamic behaviour of the cell membrane and CNTs. Advancing to the diagnosis of the cell membrane and crossing through the membrane would be part of a reliable procedure for systemic drug delivery, which would be helpful to promote the delivery performance. Systemic drug delivery provides a few methods for designing the delivery method. It provides the relation between the parameters and the effect of the final result.

In this paper, we focus on the designing parameters involved in the delivery system. Parameters changing affects the performance of drug delivery. For this purpose, analyzing the behaviour of the cell membrane is key. In this regard, we focus on molecular dynamics computing for extracting the needed force for crossing functionalized CNT through the cell membrane. Therefore, CNTs are passed in various constant velocities separately, and a frequency model is investigated to fit the recorded data. CNTs are functionalized with sodium alginate (ALG), chitosan (CHI), folic acid, and doxorubicin hydrochloride, which are mainly used in targeting the cancer cells. To provide more accurate simulation, we have also recreated a similar environment in reality. By using the MATLAB system identification toolbox, a transfer function is identified in the data. In fact, the interaction forces are the system responses, and the velocities are the inputs. Based on the frequency model, the optimal crossing time is investigated, and the solution is reported for various crossing time strategies. The cell membrane and the crossing system are modelled based on precise molecular-scale simulation. The results are useful for researchers working to implement optimal forces and trajectories delivery system. In Section 2, the overall methodology is explained; in Section 3, the molecular-scale modelling of the cell membrane and functionalized CNTs and mathematical modelling are considered. In Section 4, optimal time crossing and the criteria for proper delivery are studied. Section 5 and Section 6 are dedicated to the results and the conclusion, respectively.

## 2. Methodology

For the pipeline of the research study, there are a few steps towards the goal, which in this section will be described. In general, we have three steps: (1) crossing CNTs through the cell membrane, (2) trajectory controlling inside the cells and pointing in a correct position, (3) designing an optimal magnetic field which can support all steps for the lowest crossing time with restricted magnetic field in a healthy range. The following mentioned steps are listed below:

### 2.1. Crossing through the Cell Membrane

**Figure 1:**
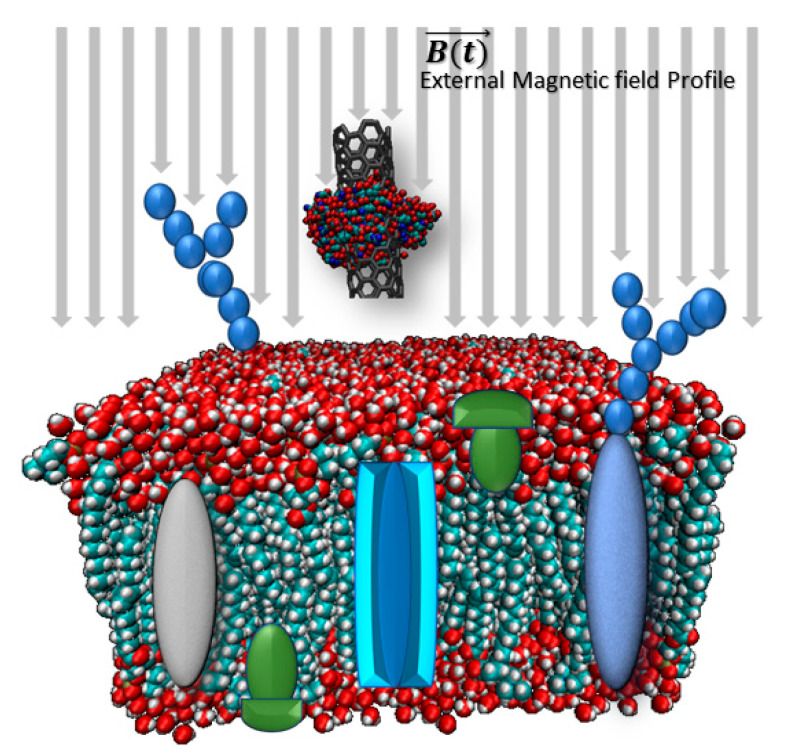
CNTs cross through membrane structure. Functionalized CNTs with drugs and magnetic nanoparticles (MNPs) are guided with a magnetic field to cross through the cell membrane. By applying a predefined external magnetic field profile on the MNPs, the exerted force leads CNTs across the membrane.

In this step, functionalized CNTs which contain nanomedicine drugs, polysaccharides, and magnetic nanoparticles (MNPs) are applied by an external magnetic field. The accurate magnetic profile causes a force on the MNPs and consequently targets the CNTs crossing through the membrane. In this step, the molecular behaviour of the cell membrane and the effect of the molecular scale of CNTs are investigated. Figure 1 shows the methodology of this step.

### 2.2. Controlling inside the Cell

**Figure 2:**
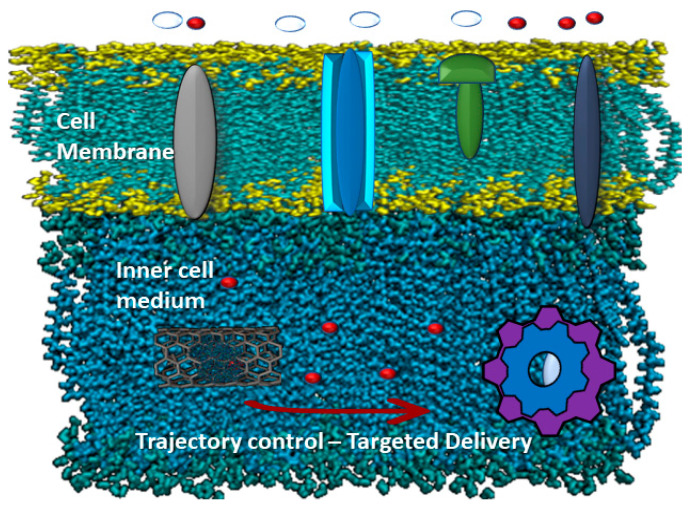
Functionalized CNTs crossing through the membrane. With a predefined magnetic field, CNTs can be guided through a desired trajectory inside the cell. The magnetic forces needed in this step are lower than those needed later, as the resistance forces are lower than they are inside the cell and cytoplasm.

In this step, as shown in Figure 2, based on the frequency changing of the magnetic field, the actuation process will be started. By increasing the frequency, a vibration of the CNTs will occur. Vibration is one step toward releasing, and the energy is converted to heat, which individually causes damage in the targeted area.

### 2.3. Profile of Magnetic Field

In this step, we review the range of magnetic fields, crossing times and energy usage in order to find if they are in the standard range. If the designed profile exceeds the maximum level of safety, the steps will be redesigned to trade off with other parameters in order to confine them in an acceptable range. Mathematical tools were used to optimize the parameters. These optimizing criteria are based on the below subjects:

- Minimum the crossing time and energy;
- Controlling the maximum peak of the magnetic field to be in a healthy range;
- Controlling the maximum peak of the magnetic gradient to be in a healthy range;
- Proper trajectory control of the drug capsule inside the cell.

The above subjects will be satisfied based on the mathematical model derived by computational analysis.

## 3. Mathematics and Modeling

In this section, the lung cell membrane and functionalized CNTs are modelled in an atomic model to investigate a proper relation between the forces and velocities in CNTs crossing through the membrane layer. The dynamics behaviour of the delivery of CNTs and drugs inside the cell is investigated, and the magnetic profile for the trajectory control and actuation of CNTs is derived. Designing the molecular scale model of various elements is described in separate subsections, and all of them are put together for a complete computational analysis.

### 3.1. Proposed Carbon NanoTube Model

**Figure 3:**
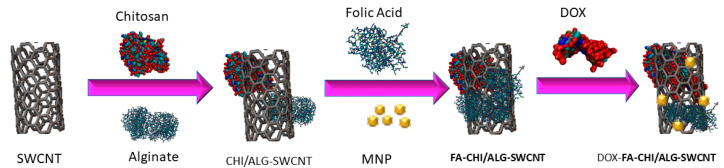
The process of conjugating a carbon nanotube with chemical compounds. Single-wall carbon nanotube (SWCNT) conjugated with sodium alginate (ALG), chitosan (CHI), folic acid, and doxorubicin hydrochloride.

**Figure 4:**
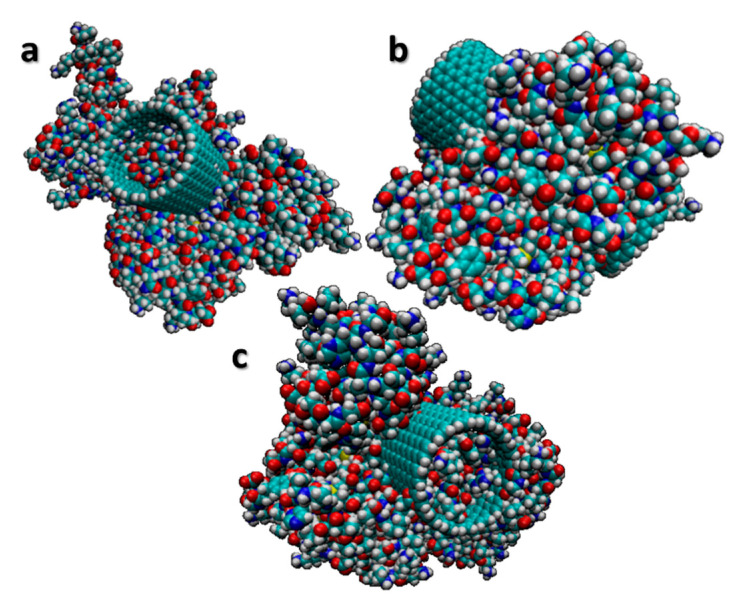
Single-wall carbon nanotube (SWCNT) conjugated with sodium alginate (ALG), chitosan (CHI), folic acid, and doxorubicin hydrochloride. (**a**–**c**) The structure in various orientations.

In the molecular-scale simulation, various shapes of carbon nanotubes are used as nanocarriers for a drug delivery application. A nanotube can be defined by two numbers (m, n). Equation (1) shows the relationship between the diameter of a CNT and (m, n) its parameters.

(1) $$d=\frac{a_{0}}{\pi }\sqrt{n^{2}+m^{2}+mn},$$

where $a_{0}$ is the lattice constant and is equal to 0.246 nm. In this study, m and n were selected to be equal to 150; based on Equation (1), the diameter would be equivalent to 20.344 nm. This study is the primary step of targeted drug delivery to cells. Therefore, the specific polymers and cancer drug (doxorubicin hydrochloride) are conjugated to the CNT walls to increase the accuracy of the simulation for the application of drug delivery to cancer cells. Figure 3 shows the process of conjugating a carbon nanotube with chemical compounds. Figure 4 shows the structure of the CNT attached to the chemical molecules in three different orientations. The MD simulation runs with various constant velocities, and pulling forces are applied to CNTs and conjugated materials to cross the whole structure through the membrane. The velocity in the simulation varies from 1 Å/ps to 2 Å/ps, with an interval 0.1 Å/ps.

### 3.2. Molecular Scale Modeling of Lung Cell Membrane

**Figure 5:**
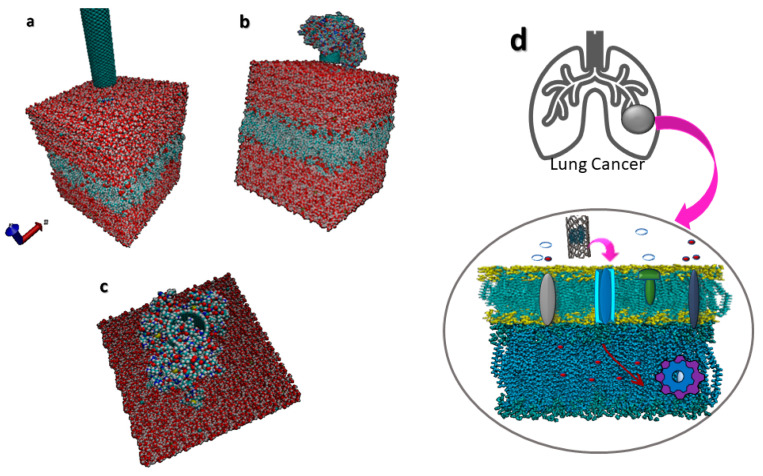
Carbon nanotube above membrane: (**a**) simple CNT above the membrane, (**b**,**c**) functionalized CNT above the cell membrane in two different orientations, (**d**) lung cell membrane and a functionalized CNT above the membrane.

A phospholipid is one class of lipids classes that plays an essential role in cell membranes and is one of the crucial components of cells. Phospholipid usually is formed in a bilayer structure. A diglyceride, a phosphate group, and choline form a phospholipid. Lipid bilayers are made of two-layer lipid molecules. In the literature, DiMyristoyl PhosphoCholine (DMPC) and Palmitoyl Oleyl Phosphatidyl Choline (POPC) are widely used in the molecular-scale simulation of membrane biological cell membrane systems. In this simulation, the POPC membrane model is selected for simulation. Figure 5 shows the functionalized and non-functionalized CNTs above the cell membrane.

### 3.3. Mathematical Modeling of Crossing through the Cell Membrane

The system identification toolbox is a collection of various mathematical algorithms to represent system models based on inputs and outputs. There are different methods to fit a frequency model based on input and output recorded data. Due to some specific behaviour of the data, a suitable algorithm will be selected. These methods are not limited to only industrial systems; they can be used in the biological and economic systems as well. Generally, there are two system identification approaches; (1) gray box and (2) Black box. The difference between the gray and black boxes is related to knowing how the system dynamics are exposed. If the understanding of the system dynamics is shallow, the black box strategies are applied based on the pre-information about the system, and the accuracy will be increased in the model by changing the number of parameters, system order, and frequency behaviour [30,31].

**Figure 6:**
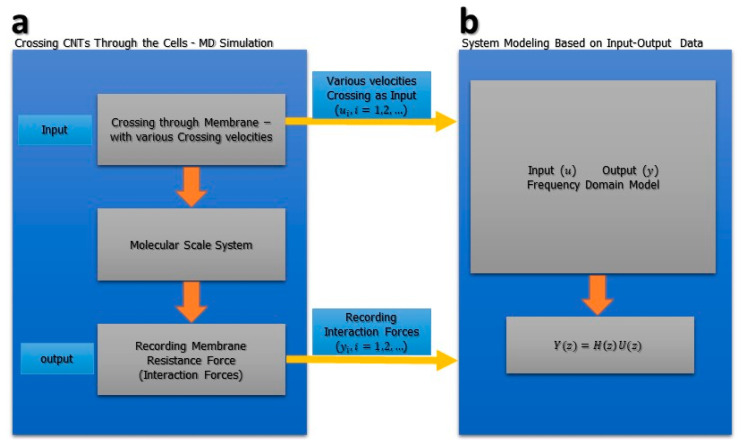
Concept of frequency domain modelling of the molecular-scale system. (**a**) Based on the designed molecular-scale model, functionalized CNTs cross through the lung cell membrane with various velocities and the interaction forces are recorded, which can be seen as a resistance force. (**b**) With the recorded interaction forces as the output and the velocity as the input, a frequency domain mathematical model is fitted on the data. The PEM system, parameters, and coefficient will be updated, and the desired parameters are extracted.

In this study, prediction error minimization (PEM) is used to estimate the system coefficients. These coefficients are located in the primary system dynamics equations. For this purpose, two steps are followed: (1) Collecting data from molecular dynamics simulation; in this step, all of the interaction forces are recorded by various velocities. (2) Initializing the parameters based on PEM and updating the parameters in order to reach the best fit. Figure 6 shows the concept design of system identification for molecular-scale systems. Equation (2) shows the mathematical equation in the frequency domain. In this equation, H is the transfer function, F is the output force, and V is the input velocity.

(2) F(z)=H(z)V(z)

### 3.4. Continuum Inner Cell Motion Dynamics: Mathematical Modeling 

**Figure 7:**
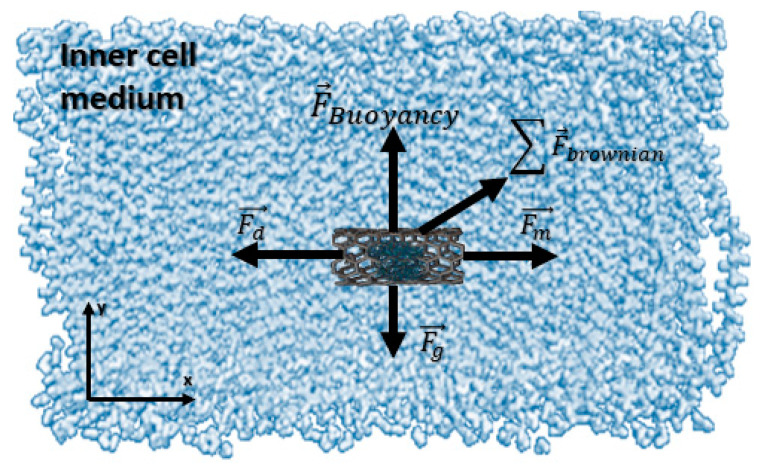
Free diagram of forces flowing inside the cell. In this diagram, the drag force is created by flow and the actuation force is applied by the external magnetic field device. Buoyancy forces and Brownian forces are shown in the figure. The total forces regarding the gravity and buoyancy are neglectable. Brownian forces create random motion, which depends on the medium temperature.

In this section, the analysis of the dynamics of functionalized CNTs is studied. After crossing the CNTs through the cell membrane, targeting the drug is the central part. For this goal, the dynamics equation is required to consider the movement of the particles. In this part, with the modelling of the particles and their motion inside the cell and the effect of the fluid forces on the particles, the translocation criteria and control of position are investigated. Figure 7 shows the force diagram applied to the CNT.

To analyze the motion of the functionalized CNTs inside the cell, the whole dynamics equation is considered. In this case, based on a Newtonian formulation, the total forces are included in the equations, which can be applied to the particles and considered the interaction forces between the particles and liquid. These forces are directly affected by the CNTs’ motions. The momentum is determined using Newton’s second law:

(3) $$\frac{d(m_{p}V_{p})}{dt}=\vec{F}$$

where $m_{p}$ is the particle mass and $\vec{F}$ is the applied force on the CNTs, which is defined as (4). The total applied force ($\vec{F}$) on the CNTs [32] consists of three components, as shown in Equation (4).

(4) $$\vec{F}=\vec{F_{m}}+\vec{F_{d}}+\vec{F_{g}}$$

$\vec{F_{d}}$ is the fluidic drag force applied by the medium on moving functionalized CNTs, and is defined as [33,34]:

(5) $$\vec{F_{d}}=\left(\frac{1}{\tau_{p}}\right)m_{p}(\vec{V_{p}}-\vec{u}),$$

where $\vec{u}$ is the velocity of the particle; $\vec{V_{p}}$ is the fluid velocity; and $\tau_{p}$ is the particle velocity response, which can be determined as [34]:

(6) $$\tau_{p}=\frac{\rho_{p}d_{p}^{2}}{18\mu },$$

where $\rho_{p}$ is the particle density and $d_{p}^{}$ is the particle diameter. Therefore, the gravitational force vector derived as:

(7) $$\vec{F_{g}}=m_{p}g\frac{\left(\rho_{p}-\rho \right)}{\rho_{p}}.$$

Regarding the direction and range of gravitational force, it can be neglected in this study. The latter is the magnetic force $\vec{F_{m}}$, which is an external magnetic field, as in Equation (8) [35].

(8) $$\vec{F_{m}}=(\vec{m}.\nabla )\vec{B}=\frac{V\Delta \chi }{\mu_{0}}(\vec{B}.\nabla )\vec{B}=\frac{V\chi_{bead}}{\mu_{0}}(b_{z}\frac{\partial b_{z}}{\partial z})$$

where $\Delta \chi =\chi -\chi_{medium}$ is the effective susceptibility of the superparamagnetic NPs relative to the intercellular, and $\mu_{0}=4\pi \times 10^{-7}\frac{N}{A^{2}}\;$ and $V=\frac{4}{3}\pi r_{ave}^{3}$ are the magnetic permeability of the vacuum and the volume of the NP, respectively. $r_{ave}^{}$ is defined as Equation (9):

(9) $$r_{ave}=\sqrt[{3}]{\frac{3}{4}L_{CNT}r_{CNT}^{2}}.$$

With a low concentration of particles, we can neglect bthe uoyancy, gravitational force, and particle interaction [36]. Therefore, using Newton’s law, we can obtain Equation (10):

(10) $$m_{p}\frac{d{\vec{v}}_{p}}{dt}=\vec{F_{m}}-\frac{18\mu }{\rho_{p}d_{p}^{2}}m_{p}({\vec{v}}_{p}-\vec{u}),$$

where $m_{p}$ and ${\vec{v}}_{p}$ are the mass and velocity of the particles, respectively. In reality, there is no flow inside the cell, and $\vec{u}$ is supposed to be equal to zero. Equation (11) shows the dynamic equations of motion:

(11) $$\begin{array}{l} m_{p}\frac{d{\vec{v}}_{p}}{dt}+\frac{18\mu }{\rho_{p}d_{p}^{2}}m_{p}({\vec{v}}_{p})=\frac{V\chi_{bead}}{\mu_{0}}(b_{z}\frac{\partial b_{z}}{\partial z})\\ \ddot{z}+\overset{\mathbb{C}}{\overset{︷}{\frac{18\mu }{\rho_{p}d_{p}^{2}}}}\dot{z}=\overset{\kappa }{\overset{︷}{\frac{V\chi_{bead}}{m_{p}\mu_{0}}}}\underset{\alpha (\alpha z+c(t))}{\underset{︸}{b_{z}\frac{\partial b_{z}}{\partial z}}} \end{array}$$

The parameters required in Equation (11) are collected in Table 1. As the magnetic field exerted by MRI can be linear along the z-axis, it is possible to suppose the magnetic field distribution, as expressed in Equation (12):

(12) $$b_{z}=\alpha z+c(t).$$

Substituting Equation (11) with Equation (12), we can obtain Equation (13):

(13) $$\ddot{z}+\mathbb{C}\dot{z}=\kappa \alpha^{2}z+\kappa \alpha .c(t).$$

Based on the derived equations, pre-defined criteria can be defined to gain the performance. According to Equation (13), c(t) is defined as the function of time, with flexibility to adjust the conditions for the position control of MNPs. Therefore, the error is defined similarly, as expressed in Equation (14).

(14) $$\begin{array}{l} e=z-z_{d}\\ \dot{e}=\dot{z}-{\dot{z}}_{d}\\ \ddot{e}=\kappa \alpha^{2}z+\kappa \alpha .c(t)-\mathbb{C}\dot{z}-{\ddot{z}}_{d}=v \end{array}$$

By setting $v=-m_{1}\dot{e}-m_{2}e$, where $m_{1},m_{2}>0$, the error dynamics would be stable, and the error tends to zero. (15) shows the criteria that satisfy the stability.

(15) $$\begin{array}{l} c(t)=\frac{\mathbb{C}\dot{z}-\kappa \alpha^{2}z+{\ddot{z}}_{d}-m_{1}\dot{e}-m_{2}e}{\kappa \alpha }\\ b_{z}=\alpha z+c(t)=\frac{\mathbb{C}\dot{z}+{\ddot{z}}_{d}-m_{1}\dot{e}-m_{2}e}{\kappa \alpha } \end{array}$$

where $l_{1}=\frac{m_{p}\mu_{0}}{V\chi_{bead}},l_{2}=\frac{6\pi \eta r_{p}\mu_{0}}{V\chi_{bead}}$,$m_{1},m_{2}>0$,$v_{p}$ is the particle velocity and α is related to the magnetic gradients in line with the z-axis. 

## 4. Optimal Designing

This section is based on the mathematical model derived described in Section 3. The optimal crossing by controlling the time of crossing is provided. In fact, the goal is to find a relation between the magnetic field and the magnetic gradient as long as the passage time is minimized. Moreover, the limitation range for the magnetic gradient and the magnetic field is also restricted to the normal range for living organs. Based on the molecular-scale calculations and the atomic model designed in previous sections, MD simulation helps us to extract the interactive force required for crossing the functionalized CNT through the membrane. Based on our nanocarrier design, the CNT contains magnetic nanoparticles in addition to drugs. The magnetic force from the external magnetic field is primarily applied to the nanoparticles attached to the nanotubes, and as a result the entire structure is translocated. A proper magnetic profile plays an essential role in minimum passage time. Equation (16) shows the mathematical relation between the magnetic field and the force. This equation generally indicates the magnetic force applied to a magnetic dipole ($\vec{m}$) in an effect of the external magnetic field ($\vec{B}$).

(16) $$\vec{F}=(\vec{m}.\nabla )\vec{B}=\frac{V\chi_{bead}}{\mu_{0}}\left(\vec{B}.\nabla \right)\vec{B}$$

In Equation (16), part of the magnetic field and gradient can be extended to Equation (17):

(17) $$\left(\vec{B}.\nabla \right)\vec{B}=\begin{array}{c} {\hat{e}}_{x}\left(b_{x}\frac{\partial b_{x}}{\partial x}+b_{y}\frac{\partial b_{x}}{\partial y}+b_{z}\frac{\partial b_{x}}{\partial z}\right)\\ +{\hat{e}}_{y}\left(b_{x}\frac{\partial b_{y}}{\partial x}+b_{y}\frac{\partial b_{y}}{\partial y}+b_{z}\frac{\partial b_{y}}{\partial z}\right)\\ +{\hat{e}}_{z}\left(b_{x}\frac{\partial b_{z}}{\partial x}+b_{y}\frac{\partial b_{z}}{\partial y}+b_{z}\frac{\partial b_{z}}{\partial z}\right) \end{array}.$$

Regarding the orientation of the functionalized CNT and the cell membrane, the applied force required for the crossing needs to be in the direction of the membrane surface normal vector. Therefore, the vector of force can be simplified to Equation (18).

(18) $$\vec{F}=\frac{V\chi_{bead}}{\mu_{0}}\left(b_{z}\frac{\partial b_{z}}{\partial z}\right){\hat{e}}_{z}.$$

By substituting the Equation (2) into Equation (18), the complete relation between the magnetic field and velocity is derived.

(19) $$b_{z}\frac{\partial b_{z}}{\partial z}=\frac{\mu_{0}}{V\chi_{bead}}{\mathbb{Z}}^{-1}[H\left(z\right)v(z)]=\frac{\mu_{0}}{V\chi_{bead}}{\mathbb{Z}}^{-1}[H\left(z\right)]{}^{}v(k)$$

In Equation (19), H is the transfer function and v is the velocity of the CNT. Since the translocation of CNT through the cell membrane is constant and equal to the thickness of the membrane, by considering the linear behaviour the crossing projection time can be expressed in Equation (20).

(20) $$\begin{array}{c} x_{membrane}\\ x_{membrane} \end{array}\}\to v_{s}=v\frac{t}{t_{s}}.$$

Moreover, the limitation boundary satisfies Equation (21):

(21) $$\begin{array}{l} L_{b}\leq b_{z}\frac{\partial b_{z}}{\partial z}\leq L_{u}\\ \{\begin{array}{c} b_{z}\frac{\partial b_{z}}{\partial z}=\overset{L_{b}}{\overset{︷}{\frac{\mu_{0}}{V\chi_{bead}}{\mathbb{Z}}^{-1}[H\left(z\right)]{}^{}v_{s_{\min}}(k)}}\\ b_{z}\frac{\partial b_{z}}{\partial z}=\overset{L_{u}}{\overset{︷}{\frac{\mu_{0}}{V\chi_{bead}}{\mathbb{Z}}^{-1}[H\left(z\right)]{}^{}v_{s_{\max}}(k)}} \end{array} \end{array}$$

On the other hand, based on the standard magnetic field generator, the range for the gradient magnetic field and the magnetic field can be defined as Equation (22).

(22) $$\begin{array}{l} 0\leq b_{z}\leq 2.4\left[T\right]\\ 0\leq \frac{\partial b_{z}}{\partial z}\leq 180\left[\frac{mT}{m}\right] \end{array}$$

Therefore, based on the frequency model, the dynamics equations and also the limitation expressed in Equation (22), the suitable area is illustrated in the result section. The curves show the criteria needed for crossing in 30 s and 1, 2, 5, and 10 min through the cell membrane.

## 5. Results

### 5.1. Results for Crossing through the Cell Membrane 

**Figure 8:**
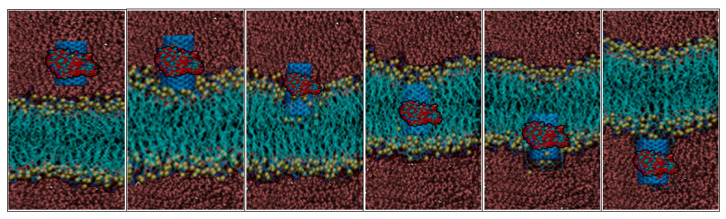
Snapshot of the crossing of functionalized CNT through the membrane. This figure shows the orientation of functionalized CNT while crossing through the membrane.

**Figure 9:**
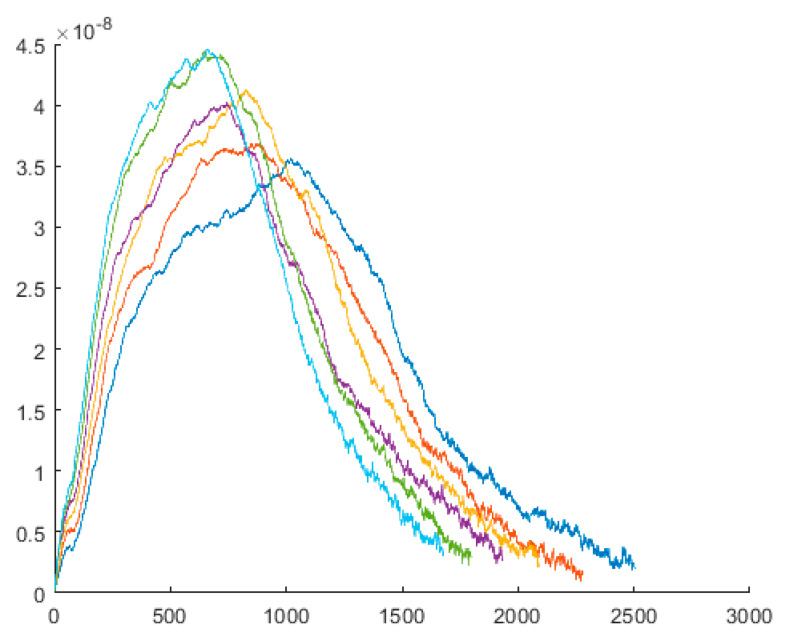
Force needed for crossing through the membrane. This shows the interaction of the force created to cross functionalized carbon nanotubes through the membrane at various velocities between 1 and 2 angstroms per second. This diagram shows an increase in the rate of passage while the crossing velocity is increased. It clearly shows that by increasing the rate of crossing, the interaction forces are increased. The X-axis is the steps, and Y-axis is the force [N].

As we discussed in previous sections, molecular-scale simulation helps us to analyze the molecular behaviour of CNT when crossing through the membrane. In this process, functionalized CNT crosses through the cell membrane, and the interaction forces are recorded. In this simulation, functionalized CNT is crossed in various velocities, and the interaction forces regarding each velocity are recorded. Figure 8 and Figure 9 shows a snapshot of crossing through the cell membrane and interaction forces vs. time, respectively.

In this research study, the input system is the impulses that indicate the velocity amplitudes. The system output is the interaction forces derived from the crossing of functionalized CNT through the cell membrane. The order of system is acquired by trial and error with the data and reaching the best fit for the recorded data. In this model, a best fit of 80% with an MSE of around 3 × 10^−8^ was achieved. Equation (2) expresses the transfer function of the system, which is a mathematical relation between the input (velocity) and output (interaction force). The parameters and the values needed for Equation (23) are collected in Table 2.

**Figure 10:**
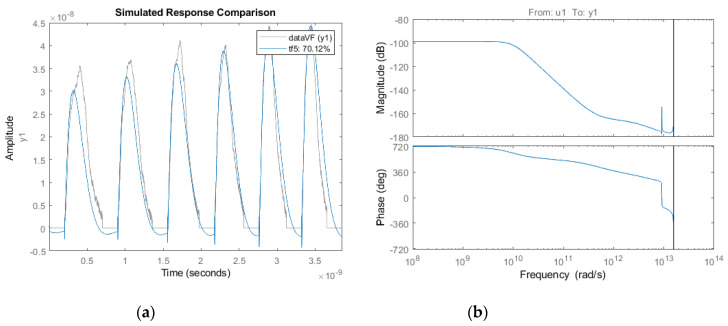
System dynamics response comparison and bode diagram. (**a**) Best fit of the system identification. In this figure, the estimated mathematical model and the real recorded data are illustrated in the same coordinate. This shows that the system behaviour and dynamics in both are similar. (**b**) Bode diagram of the identified system. In the bode diagram, there is information on the magnitude and phase vs. frequency, which provides the behaviour of the system based on frequency.

Figure 10a shows the comparable data and the best fit based on the estimated and real data, showing the similar behaviour of the system. Although the time response gives transparent information about the system behaviour, the frequency domain contains more information for analyzing the system. Figure 10b demonstrates the Bode diagram of the modelled system.

(23) $$F\left(z\right)=\frac{kz^{-1}\left(1+a_{0}z^{-1}\right)\left(1-a_{1}z^{-1}+a_{2}z^{-2}\right)\left(1+a_{3}z^{-1}+a_{4}z^{-2}\right)}{\left(1+b_{0}z^{-1}\right)\left(1-b_{1}z^{-1}\right)\left(1-b_{2}z^{-1}+b_{3}z^{-2}\right)\left(1+b_{4}z^{-1}+b_{5}z^{-2}\right)}V\left(z\right)$$

**Figure 11:**
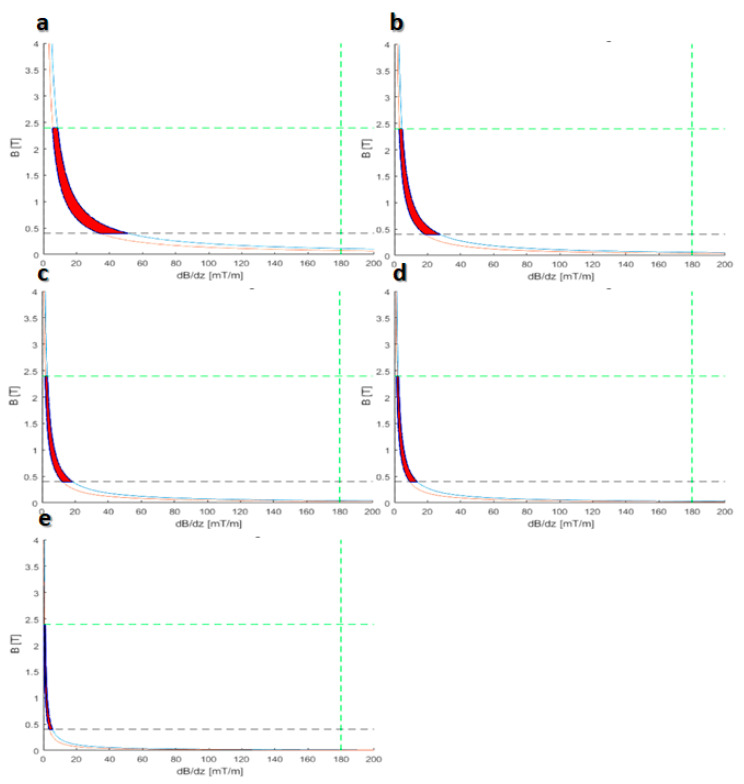
Optimal region for the crossing of functionalized CNT through the cell membrane: (**a**) region of the magnetic field and gradient for crossing within 30 s; (**b**) region of the magnetic field and gradient for crossing within 60 s; (**c**) region of the magnetic field and gradient for crossing within 90 s; (**d**) region of the magnetic field and gradient for crossing within 120 s; (**e**) region of the magnetic field and gradient for crossing within 300 s.

The results of this section are used for entering the drug to the cell. Based on the resistance force needed for crossing through the cell membrane, magnetic criteria have been designed, and the designer can control the crossing time based on the proper region illustrated in Figure 11. In the next section, which is technically post-crossing, the result of the target delivery inside the cell is discussed. 

### 5.2. Results for Inner Cells Trajectory and Position Control

**Figure 12:**
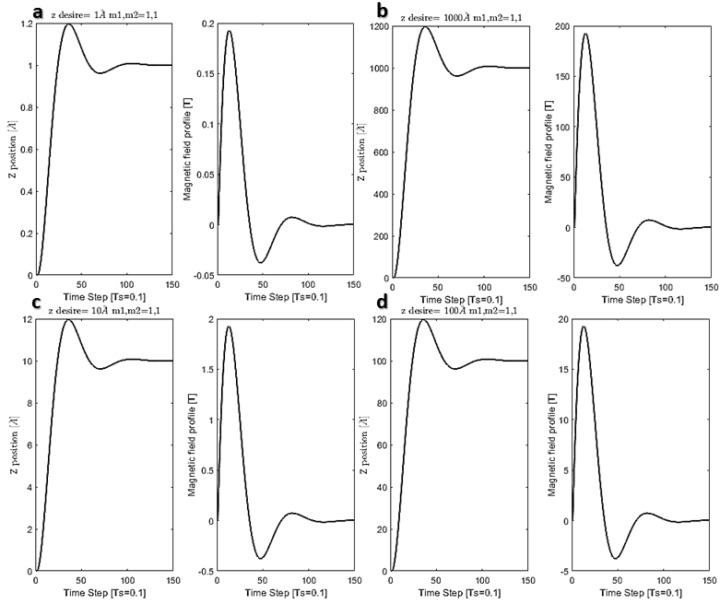
Position control of functionalized CNT inside the cell (**a**) z-position of the particle inside the cell for translocating around 1 Å and the maximum magnetic field of 200 mT. (**b**) z-position of the particle inside the cell for translocating around 1000 Å and the maximum magnetic field of 180 T. (**c**) z-position of the particle inside the cell for translocating around 10 Å and the maximum magnetic field of 180 mT. (**d**) z-position of the particle inside the cell for translocating around 100 Å and the maximum magnetic field of 18 T.

**Figure 13:**
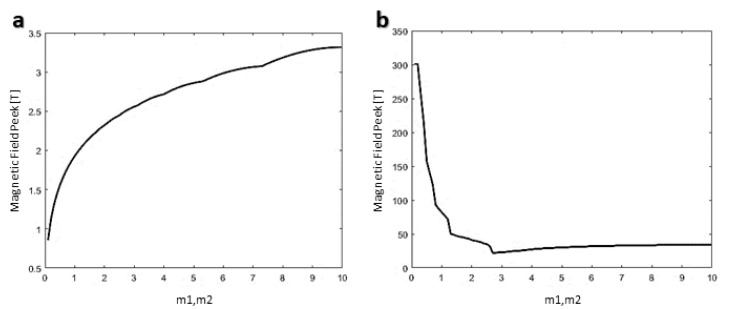
Effect of the controller parameters on the settling time and magnetic field peak. (**a**) Maximum magnetic field peak needed when the m parameters change from 0 to 10 (**b**). Settling time corresponds to m parameters, showing that, by increasing the m, settling time is reduced. Lower settling time means a higher speed of reaching the targets.

After crossing the functionalized CNT through the cell membrane, the delivery of the capsule inside the cell is the second step of the delivery. For this purpose, based on the dynamic equation derived in the previous section, the trajectory control of CNTs for the translocation of 1 Å to 1000 Å has been investigated, and the magnetic strength profile is also calculated. Figure 12 shows the magnetic field and position control of CNTs. In this figure, the translation of 1 Å and 10 Å in 5 s needs around 180 mT to 2 T of magnetic field strength. Figure 13 shows the effect of the parameters on the magnetic field peaks and settling time. Based on this figure, by reducing the settling time, which means a higher speed of targeting, the magnetic field peak is increased. In simple words, a faster delivery needs a higher magnetic field power.

## 6. Conclusions

In this study, we have analyzed and designed a drug delivery system to deliver a drug for lung cancer cells. In the proposed method, functionalized CNTs are conjugated with sodium alginate (ALG), chitosan (CHI), folic acid, and doxorubicin hydrochloride with the attachment of magnetic nanoparticles (MNPs). MNPs play the role of actuators, which can be directed by an external magnetic field. To analyze the delivery system’s behaviour crossing through the cell membrane, a molecular-scale model and MD simulation have been accomplished to extract the interaction forces needed for crossing the nanotubes through the lung cell membrane. Therefore, atomic-based model CNTs, polymers, drugs, and cell membranes were created. The complex mathematical model in the time and frequency domain has been derived from understanding the cell membrane’s behaviour, and the mathematical model was used for providing the proper criteria for crossing through the cell based on the defined time. The suitable criteria, which can be a region related to the magnetic field and magnetic gradient, have been illustrated for the crossing time of 30 s to 300 s. Moreover, based on the dynamics of CNTs and the fluid dynamic inside the cells, a suitable position control system for targeting the drug capsule inside the cell has been studied. Based on the proposed mathematical model, researchers and nanotechnologists can design a delivery system for the delivery of the drug to a specific area, which can also provide information to control the delivery time.

## Figures and Tables

**Table 1:** Parameters’ definitions.

Parameter	Definition	Value
$\mu_{0}$	the permeability of vacuum	$4\pi 10^{-7}$
V	Particle volume	$\frac{4}{3}\pi r_{ave}{}^{3}$
$\chi_{bead}$	magnetic susceptibility	0.17

**Table 2:** Dynamics system parameter values.

Parameters	Value
k	$1.7244\times 10^{-9}$
$a_{0}$	1.186
$a_{1}$	2.16
$a_{2}$	1.169
$a_{3}$	0.4392
$a_{4}$	1.06
$b_{0}$	0.9237
$b_{1}$	0.5969
$b_{2}$	1.997
$b_{3}$	0.9972
$b_{4}$	0.4635
$b_{5}$	0.9926
